# Literature Survey for In-Vivo Reynolds and Womersley Numbers of Various Arteries and Implications for Compliant In-Vitro Modelling

**DOI:** 10.1007/s13239-024-00723-4

**Published:** 2024-03-18

**Authors:** P. N. Williamson, P. D. Docherty, M. Jermy, B. M. Steven

**Affiliations:** 1 Department of Mechanical Engineering, University of Canterbury, Private Bag 4800, Christchurch, 8140 New Zealand; 2 Institute of Technical Medicine, Furtwangen University, Campus Villingen-Schwenningen, Jakob-Kienzle Strasse 17, 78054 Villingen-Schwenningen, Germany

**Keywords:** Insitu modelling, Haemodynamics, Dynamic matching, Phantom modelling, Cardiovascular research

## Abstract

**Purpose:**

In-vitro modelling can be used to investigate haemodynamics of arterial geometry and stent implants. However, in-vitro model fidelity relies on precise matching of in-vivo conditions. In pulsatile flow, velocity distribution and wall shear stress depend on compliance, and the Reynolds and Womersley numbers. However, matching such values may lead to unachievable tolerances in phantom fabrication.

**Methods:**

Published Reynolds and Womersley numbers for 14 major arteries in the human body were determined via a literature search. Preference was given to in-vivo publications but in-vitro and in-silico values were presented when in-vivo values were not found. Subsequently ascending aorta and carotid artery case studies were presented to highlight the limitations dynamic matching would apply to phantom fabrication.

**Results:**

Seven studies reported the in-vivo Reynolds and Womersley numbers for the aorta and two for the carotid artery. However, only one study each reported in-vivo numbers for the remaining ten arteries. No in-vivo data could be found for the femoral, superior mesenteric and renal arteries. Thus, information derived in-vitro and in-silico were provided instead. The ascending aorta and carotid artery models required scaling to 1.5× and 3× life-scale, respectively, to achieve dimensional tolerance restrictions. Modelling the ascending aorta with the comparatively high viscosity water/glycerine solution will lead to high pump power demands. However, all the working fluids considered could be dynamically matched with low pump demand for the carotid model.

**Conclusion:**

This paper compiles available human haemodynamic information, and highlights the paucity of information for some arteries. It also provides a method for optimal in-vitro experimental configuration.

## Introduction

### Background

Cardiovascular disease is the leading cause of death in the developed world [1]. Research on the long-term effects of surgical repair for many forms of cardiovascular disease has led to ambiguous outcomes [2]. Optical in-vitro experimentation is a growing research domain for cardiovascular disease modelling [3, 4]. Optical measurement methods such as Particle Image Velocimetry (PIV) or injection dye tracing allow for the in-vitro investigation of haemodynamic behaviour in analogous arteries and stenting [5]. PIV and injection dye tracing methods allow representative haemodynamic effects to be captured in life-like models of human arteries. The methods can also be used to predict how repair methods such as stenting or valve replacements affect arteries. Optical in-vitro experimentation does not typically use biological materials and therefore does not have the ethical obstacles that in-vivo animal or human studies have.

PIV uses a high intensity light source and camera to illuminate and capture micro-particles suspended in a working fluid. This allows the vector profile and velocity of the fluid to be determined [5]. Injection dye tracing uses a fluorescent dye that follows the streamlines of the fluid [6]. However, the outcomes of PIV analysis are limited by the suitability of the experimental conditions utilised. This is particularly important in haemodynamic modelling as the fluid structure interaction is a key element of the fluid dynamics. In particular Geoghegan et al. found that the rigid-wall assumption led to an 61% over-estimate of maximum relative wall shear stress estimation at peak systole [7]. Improving in-vitro modelling methods to better represent in-vivo conditions can provide a safer platform for haemodynamic investigation and cardiovascular implant testing prior to human trials and present a validation method to further develop computational or numerical models and simulations [3].

The physical limitations of in-vitro PIV experimentation places constraints on how haemodynamics can be modelled. In particular, phantom scaling is often required to enable robust optical capture of the experimental flow [8, 9]. This scaling requires dynamic matching of the flow characteristics to ensure the experimental flow characteristics match in-vivo behaviour [10]. Thus, certain the fluid pump characteristics must be met. Compliant phantoms are generally designed with thin walls. However, the fabrication tolerances required for precise compliance performance are difficult to achieve. This paper aims to support compliant PIV analysis experimental design by collating haemodynamic parameters of major arteries, and notes how modern PIV approaches might be applied to capture these behaviours.

### Reynolds and Womersley Number In-Vivo

In-vitro modelling often uses dimensionless scaling of fluid parameters to ensure dynamic similarity between the experiment and the physiology represented. For example, the Reynolds Number ($Re$—Eq. 1) can be used to model the viscous and momentum effects in haemodynamics, whereas the Womersley Number ($Wo$—Eq. 2) can match the time-dependent (pulsatile) behaviour in order to provide clinical significance to the experiment.

1 $$Re=\frac{4Q}{\pi D\nu }$$

2 $$Wo=\frac{D}{2}\sqrt{\frac{2\pi f}{\nu }}$$

in which $D$ is typically taken as the proximal diameter at rest [mm].

The Reynolds number is used to quantify the ratio of inertial fluid forces to viscous fluid forces. Therefore, the Reynolds Number determines the flowrate ($Q$ [m^3^ s^−1^]), that the experimental working fluid (blood substitute) must achieve to allow dimensional similarity, given the differences in kinematic viscosity ($\nu$ [m^2^ s^−1^]) between the experimental working fluid and blood. Similarly, Womersley number matching ensures the oscillatory behaviours (at frequency $f$ [Hz]) are also kinematically and dynamically matched. Ensuring dynamic similarity leads to similarity in between the flow characteristics observed experimentally and in vivo. In particular, while peak velocity and flow rate will change, the shear rate and scaled velocity profiles will be preserved (assuming Newtonian fluid). The experimentally derived shear rate can be multiplied by the in vivo dynamic viscosity to determine shear stress. Shear stress at the wall is an important factor for endothelial cell health [11–13], and is often reported in haemodynamic research.

When rigid models are used, and the working fluid is assumed incompressible, the Womersley number is often not considered. In such cases, the lack of dynamic storage potential renders the realtime modulation of the flow rate immaterial to the flow characteristics captured [14]. In such cases, the pulsatility of the flow is captured as a series of steady flow rates (e.g. [15–17]). The Strouhal number is sometimes matched in similar experiment settings. However, the Strouhal number is a function of the Reynolds and Womersley numbers, so will be matched if these metrics are matched.

The haemodynamic pressure and flowrate waveforms change throughout the body [18]. In-vitro analysis of arteries is typically segmental, meaning the cardiac waveform for the specific artery being analysed must be isolated. In haemodynamic modelling, it is important to determine the Reynolds and Womersley Numbers for each specific artery to enable dimensional similarity between the in-vivo artery and in-vitro model. However, there is no large, current, and publicly available data bank with the Reynolds Number and Womersley Number ranges for the main arteries in the human body. As such, finding these parameters for arteries is not always possible. Canine data has been compiled [19]. However, canine Reynolds and Womersley Numbers are not necessarily indicative of human numbers [20] and canine waveforms in human geometry may not preserve the clinical significance of in-vitro experimentation. Some studies also have also used parameters for porcine arteries due to their physiological similarities to human arteries [21].

### Compliant Phantoms In-Vitro

Optical in-vitro modelling methods are continually improving with new flow circuits designs and production of more complex and representative arterial geometries. Compliant modelling is increasingly being used as studies have shown the importance of compliance in arteries such as the carotid, brachiocephalic and the aorta [9, 22–26]. Rigid modelling can have a tendency to over-predict wall shear stresses and under-predict recirculation or retrograde flow [7, 9]. These flow characteristics can have a significant effect on the growth patterns of the endothelial cells that line the artery and therefore on the regeneration of the artery [27–29]. Thus, compliant artery models may provide a method of safe investigation into the potential for deleterious flow behaviours caused by candidate stents.

However, optical modelling techniques require optically transparent materials for phantom artery manufacture to capture fluid flow. Hence, in-vitro phantom materials are typically optically transparent [3]. In some rigid-wall studies, glass has also been used [3]. However, for compliant models, elastomers are required. The most common elastomer used in in-vitro arterial modelling is Sylgard 184 (Dow Corning, Midland, MI, USA) [3, 23, 30]. Sylgard 184 is a two part silicone elastomer consisting of a predominantly Polydimethylsiloxane base and curing agent [31–33]. Sylgard 184 has a Young’s Modulus of 1.32 MPa and refractive index of 1.403 when cured at 23 °C. However, the Young’s modulus of Sylgard 184 does not match the Young’s modulus of healthy thoracic arteries (approximately 0.53 compared to 0.70 MPa, respectively [8]). In regions of stenosis, compliance is much lower [34], and in regions of delamination or aneurysm, compliance is typically higher [35]. In arteries, normalised compliance is a measure of arterial distensibility and defines the artery wall deformation induced volume change in response to pulse pressure [36]. Transparent elastomer materials are typically matched via normalised compliance (Eq. 3) [37];

3 $$C=\frac{1}{A}\bullet \frac{dA}{dP}\approx \frac{4}{\pi {D}^{2}}\bullet \frac{\pi {D}^{3}}{4Eh}=\frac{D}{Eh}$$

In which, $C$ is normalised compliance [MPa^−1^], $A$ is the cross-sectional area [mm^2^], $D$ is diameter [mm], $E$ is the Youngs Modulus [MPa] and $h$ is the wall thickness [mm].

Compliant model construction is limited by manufacturability of phantom arteries with suitable wall thickness. To date, a phantom artery with a 1 mm (± 10%) wall thickness represents the smallest consistent wall thickness reported [9]. Thinner walls tend to have higher proportional wall variability and are prone to uneven expansion or rupture [9]. To achieve appropriate wall thicknesses within fabrication limitations, models typically have to be scaled up to greater than life-scale. Scaling up the phantom requires scaling up the flowrate of the working fluid (commonly aqueous). Increasing flow may also increase pressure demands which may not be a cost effective solution for experimental models of large arteries, such as the aorta. Rigid-wall models are not dependent on the lower limit of wall thickness manufacturability and therefore can readily be manufactured at life-scale or smaller for more cost-effective experimentation.

### Working Fluid Analogues

**Fig. 1:**
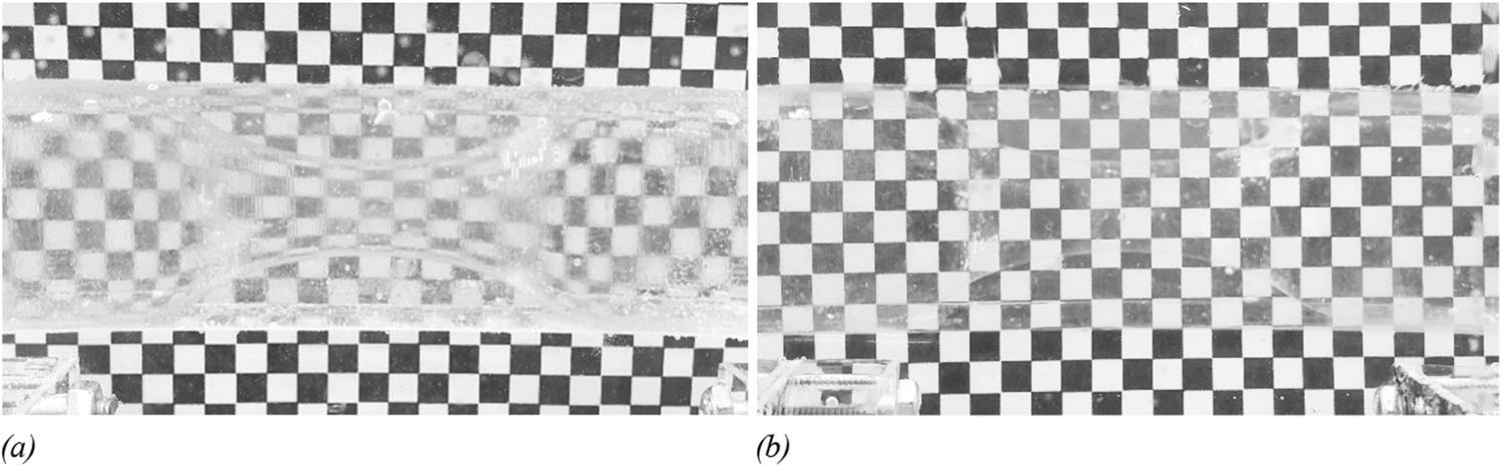
**a** Unmatched refractive index, **b** matched refractive index

The working fluid presents another design limitation of experimental modelling using a compliant phantom. The working fluid must be transparent and refractive index matched to the elastomer used in the phantom. Refractive matching removes light distortion through a model, thereby making the model almost invisible when filled with the working fluid (Fig. 1). The most common mixture used is a water–glycerine solution [3, 38, 39]. The ratio of water to glycerine can be adjusted to obtain a refractive index match to the elastomer, a similar density to blood. Furthermore, the mixtures are easily maintained and reasonably inexpensive (glycerine costing $21.08 USD per kilogram). However, the kinematic viscosity of the popular 40/60 water–glycerine ratio is nearly three times that of blood (Table 1).

**Table 1:** Properties of transparent blood analogues (W = water, Gly = glycerine, NaI = sodium iodide, U = urea)

Fluid	Density $${\varvec{\rho}}$$ (kg m^−3^)	Dynamic viscosity $${\varvec{\mu}}$$ ($$\times$$ 10^−3^ Pa s)	Kinematic viscosity $${\varvec{\nu}}$$ ($$\times$$ 10^−6^ m^2^ s^−1^)	Refractive index	Cost per KG (US $)^a^
Blood	1060	3.80	3.58	Opaque	N/A
40/60 W-Gly [3]	1156	10.8	9.34	1.41	12.83
46/29/25 W-Gly-U [39]	1130	3.56	3.15	1.41	11.00
46/29/25 W-Gly-NaI [39]	1229	3.12	2.53	1.41	86.10

^a^Prices obtained from Carolina Biological Supply Company (NC, USA), laboratory grade

Yazdi et al. [3] conducted a review of current PIV modelling techniques, in which numerous working solutions were identified. The results from Yazdi et al. [3] are summarised in Table 1. Commonly, sodium iodide is included in an aqueous glycerine mixture to raise the refractive index without significantly raising the viscosity. However, sodium iodide has a high cost of around $320 USD per kilogram and some associated health risks [39]. Another alternative uses urea to raise the refractive index of a water and glycerine mixture. Urea is significantly cheaper, costing $19.80 USD per kilogram and has a lower health risk rating [39].

## Methodology

This paper will determine the range of Reynolds and Womersley numbers for haemodynamics in healthy human subjects reported in literature and show how these values influence in situ modelling of haemodynamics.

### Part 1: Literature Search

A literature search was conducted on the peak Reynolds and Womersley numbers of 14 key arteries in the human body: ascending aorta, aortic arch, descending aorta, suprarenal aorta, infrarenal aorta, common iliac, femoral, brachiocephalic, subclavian, carotid, renal, superior mesenteric, basilar and internal carotid arteries (ICA). The peak Reynolds Number was selected for investigation, as it indicated the maximum flowrate that must be achieved to maintain physiological accuracy. Multiple search engines were used including Google Scholar, ResearchGate and PubMed. “Reynolds Number”, “Womersley Number”, “In-vivo” and each of the 14 arteries were used as keywords for each search. Of these only 10 articles provided novel in-vivo information specific to the human body. Ranges for the peak Reynolds and Womersley numbers were collected from in-vitro or in-silico sources when an in-vivo source could not be found. The in-vitro and in-silico were accepted provided that the outputted data values were obtained using in-vivo inputs. In-vitro and in-silico data was also compared against the in-vivo ranges of peak Reynolds and Womersley number values for sections of the aorta. The overall number of sources found with information on the peak Reynolds number and Womersley number was 21. Data was compiled by type and the range of peak Reynolds and Womersley Numbers were averaged (Table 4). The standard deviation for the Reynolds number was calculated based on the overall population of each source and provided as a range in Table 4. Symmetry was assumed between left and right variants of the common iliac, femoral and subclavian arteries. In reality, there are geometric, flow and, therefore Reynolds and Womersley number differences across the left and right variants of these arteries. However, the intra-artery differences were assumed negligible compared to the inter-artery differences. Due to the paucity of available sources, data was compiled from studies of normal healthy arteries as well as irregular geometries or diseased states. A range of values, derived from multiple sources, and were typically provided for each artery.

### Part 2: Case Studies

Two case studies were carried out comparing three working solutions against different scale models manufactured from Sylgard 184. The first case study investigated in-vitro modelling an ascending aorta geometry. The ascending aorta is the largest, most compliant artery in the human body and is subject to the highest Reynolds and Womersley numbers in the arterial system (Table 4). This makes it difficult to mimic in-vitro.

The second case study focuses on the left common carotid artery. This artery was selected as it is much smaller and less compliant than the ascending aorta, but also had a good basis of in-vivo information available to inform the model. Though it is not smallest artery considered, it represents the other end of the spectrum for modelling compared to the ascending aorta. The in-vivo geometry parameters and compliance ranges used for the case studies of each artery are provided in Table 2.

**Table 2:** Structural parameters for the in-vivo ascending aorta and left common carotid artery

	Young’s modulus (MPa)	Average diameter at rest (mm)	Compliance range (MPa^−1^)
Ascending aorta	0.526	32.0 [40]	29.0–37.7
Left common carotid	0.700	8.00 [8]	16.8–19.3

Normalised compliance as a function of wall thickness was calculated for Sylgard 184 silicone using the diameter provided in Table 2 at 1×, 1.5× and 2× life-scale for the ascending aorta and 1×, 3× and 5× life-scale for the common carotid artery (Eq. 3). The curves were then plotted against the known compliance ranges of the two arteries under investigation [37]. The curves were analysed to determine which scales were or were not capable of being manufactured using Sylgard 184. 1 mm was used as the minimum manufacturable wall thickness based on this being the smallest recorded consistently manufactured wall thickness using lost-core casting [9].

The optimal in-vitro model configuration was selected as the model with the lowest required pump power. The power was inferred based on flowrate and time requirements for physiological matching. The Reynolds and Womersley numbers were linked using diameter (*D*) and kinematic viscosity (*ν*). Four equations were produced, as shown in Table 3.

**Table 3:** Equations 4a–d formation

	For diameter	For viscosity
Rearrange ${\varvec{R}}{\varvec{e}}$	$\to D=\frac{4Q}{\pi Re\nu }$	$\to \nu =\frac{4Q}{\pi ReD}$
Substitute into ${\varvec{W}}{\varvec{o}}$	$Wo=\frac{2Q}{\pi Re\nu }\sqrt{\frac{2\pi f}{\nu }}$	$Wo=\frac{D}{2}\sqrt{\frac{2{\pi }^{2}fRe}{4Q}}$
Rearrange for ${\varvec{Q}}$	$Q=\sqrt{\frac{W{o}^{2}R{e}^{2}\pi {\nu }^{3}}{8f}}$	$Q=\frac{{\pi }^{2}fRe{D}^{3}}{8W{o}^{2}}$
Optimise for the largest range produced Eq. 4a–d	(a) $Q=\sqrt{\frac{W{o}_{max}^{2}R{e}_{max}^{2}\pi {\nu }^{3}}{8f}}$	(c) $Q=\frac{{\pi }^{2}fR{e}_{max}{D}^{3}}{8W{o}_{min}^{2}}$
	(b) $Q=\sqrt{\frac{W{o}_{min}^{2}R{e}_{min}^{2}\pi {\nu }^{3}}{8f}}$	(d) $Q=\frac{{\pi }^{2}fR{e}_{min}{D}^{3}}{8W{o}_{max}^{2}}$

The four equations were plotted in Figs. 4 and 6 for each working solution viscosity (Eq. 4a, b) and scale diameter (Eq. 4c, d).

Blood was assumed Newtonian in the ascending aorta as the concentration on red blood cells in the bloodstream is less than 10% [18]. Non-Newtonian blood analogues were ignored in this study as the primary focus was on determining a method to model the largest and most compliant artery in the human body, and the effects of shear thinning are significant predominantly in smaller arteries where the concentration of red blood cells exceeds 10% of the overall fluid [18].

## Results

### Part 1: Literature Search

The ranges for the minimum and maximum peak Reynolds and Womersley numbers for each of the 14 arteries were combined and reported in Table 4 along with their data type and source. There was a larger pool of primary sources to collect information from for the segments of the aorta. However, there was very limited information on the smaller arteries such as the femoral, renal and superior mesenteric. The in-vivo Reynolds numbers for the sections of the aorta were typically reported higher than those used in in-vitro or in-silico studies, meanwhile the Womersley Numbers were typically lower.

**Table 4:** Reynolds and Womersley Numbers for 14 key arteries

Artery	Min Re_peak_	Min Re_peak_ range (S.D.)	Max Re_peak_	Max Re_peak_ range (S.D.)	Min Wo	Max Wo	Data type	Sources	Notes
Ascending aorta	3144	1357	6836	1570	15.0	20.6	In-vivo	[41–45]	[41] includes elderly
	2970		3302		19.3	22.7	In-vitro and in-silico	[36, 40, 46–48]	[40] tortuous,
Aortic arch	3318	176	5733	1102	13.3	17.2	In-vivo	[42, 49]	[49] adult & DAA child
	2431		2666		14.5	15.9	In-vitro and in-silico	[40, 50, 51]	[40] aged, irregular; [50] dissection
Descending aorta	2728	867	4805	289	11.2	14.2	In-vivo	[41, 42, 44]	
	1169		2196		11.1	15.9	In-silico	[40, 46]	[40] aged, irregular
Suprarenal aorta	2000	N/A	6000	N/A	12	12	In-vivo	[41]	Includes elderly
Infrarenal aorta	1324	403	3536	1063	12	12	In-vivo	[41, 52]	[41] includes elderly
Common iliac	390	N/A	620	N/A	*7.7*	*12*	In-vivo	[52]	
Femoral	980		980		7.74	7.74	In-vitro	[53]	
Brachiocephalic	720	N/A	1080	N/A	8.2	9.8	In-vivo	[44]	
Subclavian	720	N/A	880	N/A	6.8	8.1	In-vivo	[44]	
Common carotid	556	129	716	72	5.66	6.46	In-vivo	[44, 54]	
	750		766		4.51	4.51	In-vitro and In-silico	[36, 55, 56]	[55] middle-aged
Renal	277	N/A	900	N/A	6.6	6.6	In-vitro	[57]	
Superior mesenteric	12	N/A	200	N/A	5.27	5.27	In-silico	[46, 58]	[58] dissection
Basilar	150	N/A	500	N/A	2.73	2.73	In-vivo	[59]	Aneurysm
ICA	150	N/A	300	N/A	5.33	5.33	In-vivo	[59]	Aneurysm

Italics = inferred values based on surrounding data. Unless noted, data from either undefined, or young and healthy participants was obtained

**Fig. 2:**
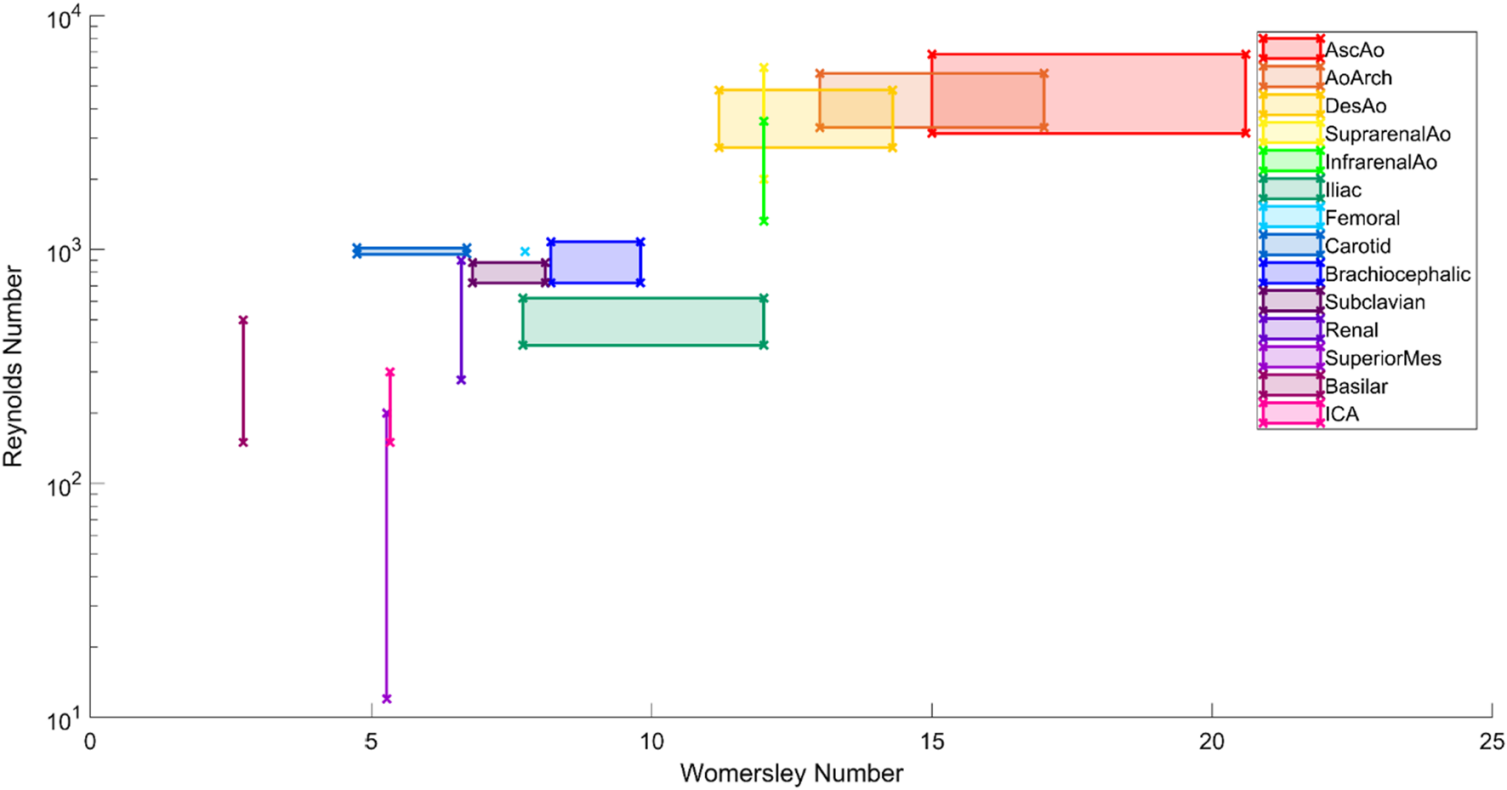
Reynolds and Womersley for 14 main arteries

Figure 2 presents the tabulated in-vivo data. Where in-vivo data was not available in-vitro or in-silico measurements were plotted (as accepted in Table 4). The proximal arteries such as sections of the aorta had significantly higher Reynolds and Womersley numbers. The reported in-vivo ranges were also much larger than distal arteries showing that inter-patient variability must be higher in the larger, more compliant, arteries.

#### Case Study: 1—Ascending Aorta

**Fig. 3:**
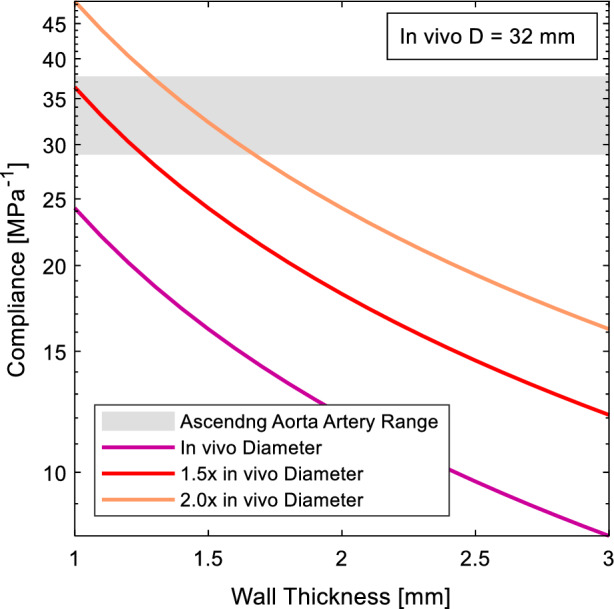
Model compliance for variable wall thickness of Sylgard 184 for different scales of the ascending aorta diameter

The compliance for each scale model manufactured from Sylgard 184 was calculated based on the wall thickness and the diameter scale. Figure 3 shows that a life-scale model cannot be reliably manufactured to match the compliance of the human ascending aorta, as it falls below the minimum wall thickness requirement of 1 mm. However, both 1.5× and 2× life-scale models can be reliably manufactured.

**Fig. 4:**
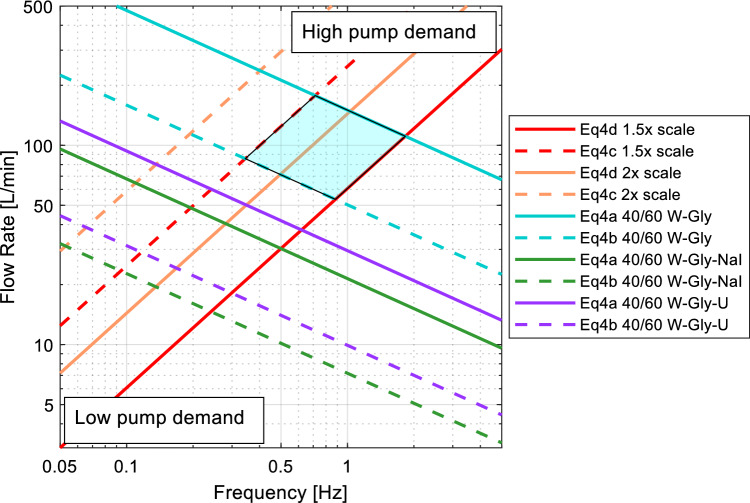
Flowrate and frequency comparison of ascending aorta model scales and working solutions for in-vitro experimental set-up (W = water, Gly = glycerine, NaI = sodium iodide, U = urea). The shaded area shows the possible experimental domain when modelling the ascending aorta with a 1.5× scale and a water–glycerine working fluid

Three potential working solutions were investigated to determine the flowrate and frequency requirements to achieve known ranges of peak Reynolds and Womersley Numbers for each of the feasible model scales identified in Fig. 3. The lines of the Fig. 4 denote fixed diameter curves and fixed viscosity curves for the maximum (solid lines) and minimum (dashed lines) Reynolds and Womersley numbers. The domain between the intersections of these curves can generate a range within which the Reynolds and Womersley numbers are representative of the ascending aorta (Fig. 4). Six allowable ranges were identified, represented by the shaded boxes. Figure 4 indicates that a 1.5× life-scale model would require a peak flowrate of between 27 and 91 L min^−1^ using a water, glycerine and sodium iodide solution. However, significant overlap can be seen between the sodium iodide solution (green) and the urea solution (purple). To fit within the Womersley and Reynolds range of the ascending aorta, a 60/40 water–glycerine working fluid (blue) has a minimum flow rate of 53.5 L min^−1^ and maximum flowrate of 178 L min^−1^ for the smallest manufacturable model (1.5×).

#### Case Study: 2—Left Common Carotid Artery

**Fig. 5:**
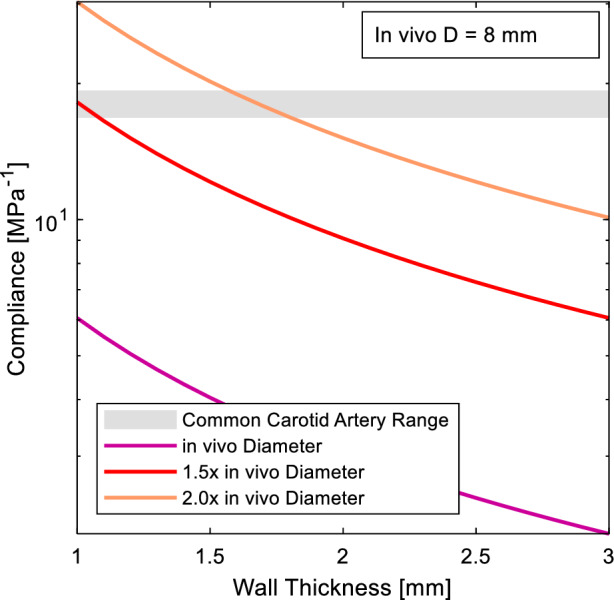
Model compliance for variable wall thickness of Sylgard 184 for different scales of the common carotid artery diameter

The compliance for each scale of the left common carotid artery is presented in Fig. 5. A life-scale model cannot be reliably manufactured to match the compliance of the common carotid artery. However, both 3× and 5× life-scale models can achieve the common carotid artery compliance. The range of allowable wall thicknesses are presented in Table 5.

**Table 5:**
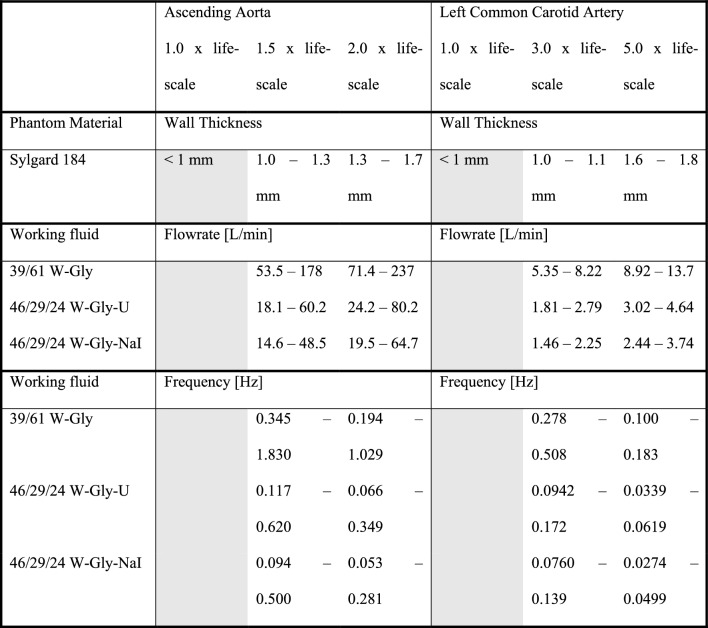
Summary of ascending aorta and common carotid artery case study outcomes

Greyed out boxes indicate non-achievable models (W = water, Gly = glycerine, NaI = sodium iodide, U = urea)

**Fig. 6:**
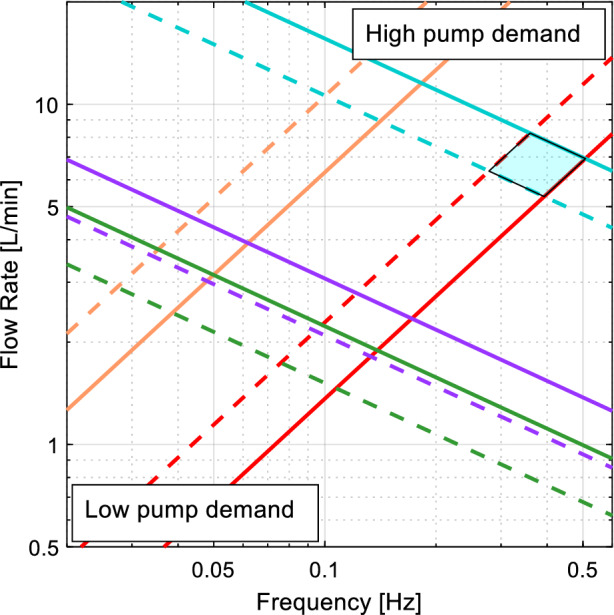
Flowrate and frequency comparison of common carotid artery model scales and working solutions for in-vitro experimental set-up. Please refer to the Figure 4 legend

The three potential working solutions were compared against the model scale to determine the six potential setups for modelling a carotid artery in-vitro, highlighted by the shaded regions. Figure 6 indicates that increasing the life-scale above 3× leads to a low flow pulse frequency and thus long cycle period. The peak flowrate of the water–glycerine solution (blue) was 13.7 L min^−1^ which was smaller than the lowest required flowrate for the ascending aorta model. The overlap between solutions is significantly less than for the ascending aorta model.

The limits of wall thickness, and bounds of volumetric flowrate and pulse frequency, as determined in case study 1 and 2 for the ascending aorta and common carotid artery as summarised in Table 5. Where the criteria for manufacturability was not met, the entire column was greyed out and the remaining potential solutions discussed.

## Discussion

### Part 1: Literature Search

Precise and relevant experimental modelling of haemodynamic flow in human arteries is reliant on precise mimicry of in-vivo dynamic fluid properties and realistic fluid-structure interactions. A literature search for Reynolds and Womersley Numbers of 14 specific arteries in the human body showed a paucity of information for some arteries, despite their clinical interest. The Reynolds and Womersley numbers for various arteries have been complied for canines [19]. The values for smaller arteries have also been provided for other animals such as pigs, rabbits and horses [19]. However, improved clinical relevance relies on knowledge of human haemodynamic parameters. Table 4 shows that there was rich information on haemodynamics in each section of the aorta. The common carotid artery was also well researched. However, there was a lack of data regarding the arteries that branch from the aorta.

Many of the important arteries, such as the femoral, renal and superior mesenteric arteries did not yield any in-vivo Reynolds or Womersley number values. The lack of in-vivo information can make modelling impossible without assumptions that may not have been validated and therefore reduce the clinical applicability of the results obtained. The input data from in-silico and in-vitro studies were included when in-vivo data could not be located [46, 52, 57, 60]. Since the domains of these arteries shown in Fig. 2 seem like sensible extrapolations from the numbers of proximal arteries, it was assumed these values could be trusted. However, this may be expected as the in-vitro/in-silico numbers were derived as functions of in-vivo numbers. The Reynolds and Womersley numbers of the femoral, and superior mesenteric arteries are similar to similar sized arteries. However, the range of in-vivo values of the renal artery is comparably large. While these studies may provide a good indication of the likely Reynolds and Womersley, direct in-vivo validation would be beneficial.

Search results for in-vivo Reynolds and Womersley numbers will commonly present canine data collated by Caro [19]. However, canines typically have lower Reynolds and Womersley numbers than human subjects. For example, searching “Abdominal Aorta Womersley number” will return Wo = 8 as the result, linked to the ‘Womersley number’ Wikipedia page [61]. Following the citation path indicates that this Womersley number was determined for a canine abdominal aorta at 2 Hz, meanwhile the human abdominal aorta Womersley number is typically 12 (Table 4). Failing to note this discrepancy will lead to inappropriate dynamic matching and therefore unrepresentative results and recommendations from simulation and experimentation.

There were notable discrepancies between the measured in-vivo and selected in-vitro Reynolds and Womersley numbers. Table 4 shows that in-vitro and in-silico Reynolds numbers were typically lower than in-vivo. This discrepancy suggests that experiments tended to use lower flowrates than may be expected in reality. Achieving the appropriate flowrate may be difficult in large arteries such as the ascending aorta, particularly when using blood analogues that have a higher kinematic viscosity than blood. However, the Womersley number tended to be reported higher in-vitro and in-silico than in-vivo. The higher Womersley was most notable in the ascending aorta which may be accounted for by model scaling in in-vitro studies [9, 23]. The Womersley number is proportional to diameter (Eq. 2), and therefore scaling models larger, without compensating for time period, leads to a higher Womersley number and lower Reynolds number. Typically, lower Reynolds numbers are accepted in-vitro due to the large range of potential peak Reynolds numbers presented across papers and the large standard deviations. In particular, Fig. 2 indicates there is large variability in Reynolds numbers in the human aorta.

In some in-vitro and in-silico investigations, a time-averaged or mean Reynolds number was presented despite modelling pulsatile flow [55, 62]. The diameter of an artery or compliant model increases at peak systole and makes determining an accurate peak Reynolds number more difficult, and therefore providing the mean Reynolds may be more accepted. However, using the mean Reynolds number to characterise a flow may result in misidentification of turbulent flow as laminar as the mean Reynolds number is typically 3–4× smaller than the peak Reynolds [42]. Some investigations purposely utilise steady-state conditions to determine the basic haemodynamic effects of cardiovascular implants [63, 64]. For example, Liepsch et al. [57] showed that steady flows may identify some haemodynamic anomalies which are caused by features of the arterial geometry. However, there is evidence that pulsatile flow induces regions of turbulence at much lower Reynolds number than is generally accepted as the turbulent threshold [65, 66].

### Part 2: Case Studies

The trend across Figs. 3, 4, 5, and 6 is that increasing the scale of the model increases the wall thickness, flowrate and pulse frequency required to achieve a physiological model. The aorta is one of the more well studied arteries, as evident by the much high incidence of in-vivo information obtained (Table 3). However, as the case study of the ascending aorta indicates, sections of the aorta can be some of the hardest arteries to experimentally model when trying to achieve a compliance matched, physiological model. For an ascending aorta model, a life-scale model cannot be manufactured from Sylgard 184 silicone. Sylgard 184 could be used to make a 1.5–2× life-scale model of the ascending aorta or a 3–5× life-scale model of the common carotid artery.

When modelling the ascending aorta, a water and glycerine working solution consistently required a volumetric flowrate three times greater for than the urea or sodium iodide solutions for the same Reynolds number. The lowest allowable flowrate for the water and glycerine mixture had a 4.3% overlap with the highest flowrates allowable using the urea mixture. This means that a much higher pump power is needed for to achieve physiologically matched waveforms. Furthermore, a frequencies of more than approximately 1 Hz is required for the water and glycerine mixture. The higher pulse frequency requires higher pump resolution to precisely match the input waveform. The working solution with sodium iodide has the lowest flowrate and lowest pulse frequency, irrespective of diameter. As a three-part working solution, it may be harder to maintain the solution long-term due to disproportionate evaporation rates. However, careful storage and maintenance of the solution could ameliorate this issue. Sodium iodide has associated health risks to consider and costs more than glycerine and urea (Table 1). There is a 66-67% overlap in the flowrates and a 73% overlap in frequency of the waveform between the sodium iodide and urea solutions to achieve the same Reynolds and Womersley numbers. This implies similar pump demands across the working fluids. The urea solution is a three-part solution with the same water, glycerine and salt ratio (by mass) as the sodium iodide solution. Urea also has a lower health risk rating. As such it may be a suitable solution for modelling large arteries such as the aorta.

There was less overlap in the flowrates and frequencies for the different working solutions of the common carotid artery model. The urea and sodium iodide solutions only overlapped by 33% in flowrate and 47% in frequency. The peak flowrate for the sodium iodide solution was 3.74 L min^−1^ for a model that is 5× life-size and would require a time period of at least 20 s per waveform. A waveform with such a long time period would not be efficient for ensemble PIV measurement where multiple image pairs are required for temporal or spatial averaging [5]. For injection dye tracing, the slow flow, long waveform could be beneficial to capture high-resolution fluid motion. The urea solution could achieve the same Reynolds and Womersley numbers with a slightly higher flow and shorter period so may allow a more efficient experimental setup. The water and glycerine two-part mixture required a maximum flow of 13.7 L min^−1^ for the 5× life-scale model. Despite being over four times higher than the flow required for the sodium iodide solution, the flowrate is still lower than the smallest needed flowrate for the ascending aorta model. Thus, achieving flowrates required to model the common carotid artery is easier than the aorta. The waveform for the 5× life-scale model required a time period of at least 5.46 s for Womersley matching. A 5.46 s waveform duration may be more suitable for experimental set ups that require high repeatability such as ensemble PIV analysis [5].

A water and glycerine working solution is an inexpensive, easily mixed and easily maintained two-part working solution that is refractive matched at a mass ratio of 40:60. However, for modelling the largest artery in the human body, the pump demands are high, and it is potentially difficult to achieve Reynolds and Womersley number matching (Fig. 4). A trade-off between achieving Reynolds or Womersley number matching is often necessary when using water and glycerine solutions in large arteries models. Whilst there are many pumping systems capable of achieving flows over 237 L min^−1^, they rarely have the response time to change direction, accelerate and decelerate within the time period required to also meet the Womersley parameters. It may be possible to design and manufacture a piston pump with powerful enough actuators to provide the appropriate flow within required time period, however, changing the working solution would provide a more cost-effective solution.

Rigid models do not have the limitations induced by the precision demands of thin-walled phantom fabrication. In such cases, models can use a simple lost-core casting technique [67, 68]. Such experiments are limited by the field of view possible in the PIV camera setup, the pump power, and the cost of fabrication materials [5]. These constraints are more lenient on what is possible. However, rigid models can fail to capture recirculation, retrograde flow, and other flow characteristics that arise from lumen distortion [22, 69].

### Limitations

This paper focused on only three Newtonian transparent blood analogues. The water and glycerine two-part mixture is the most commonly used working fluid in in-vitro experimentation [9, 23, 39, 70] and sodium iodide is a common additive [39]. The three part solution with urea is not as common. However, urea solutions are becoming more popular after Brindise et al. determined its behaviour is very similar to sodium iodide [39]. Non-Newtonian transparent blood analogues were not considered in this investigation as the focus was on experimental modelling of larger arteries. Non-Newtonian behaviour becomes more important when modelling smaller arteries or capillaries as the red blood cells have a greater effect on the fluid flow [18]. Transparent blood analogues that could not achieve a refractive index between 1.41 and 1.43 were also ignored as this is the common refractive index range for elastomers, and refractive index matching is required to avoid optical distortion in PIV experimentation and dye tracing.

There are also many considerations in haemodynamic modelling that were not directly considered in this paper. In particular, there was no consideration for transverse motion of arteries. This effect is notable in large arteries with significant curvature [69]. Furthermore, stenosis, delamination, and aneurysm have significant effects on localised arterial compliance. While the increased stiffness of stenosis is quite simple to replicate in vitro, the increased compliance in areas of delamination or stenosis will require thinner walls. These regions of thin walls are particularly difficult to fabricate with confidence. The modeller must also consider how generic they wish their model to be. More generic models may yield more generalizable results. However, such results will not be directly representative of any particular individual.

## Conclusions

The main limitation of in-vitro and in-silico modelling is the paucity of in-vivo information for many important arteries within the human body. The in-vitro case studies presented in this paper show that there are possible modelling methods that enable large and small arteries to be modelled using surrogate models and transparent blood analogues. However, the lack of information for some arteries requires potentially incorrect assumptions to estimate Reynolds or Womersley numbers. These assumptions may lead to results that are not physiologically representative. This research also identified a simple method for designing experimental setups required to model haemodynamics in certain arteries. For example, it was shown that effectively and economically mimicking arterial compliance and the high flow and frequency demands of the large arteries required a three-part working fluid.

## Data Availability

All data used in this analysis was derived from the literature, and conclusions drawn from analysis of relevant equations. Codes to generate content in the paper can be made available to interested parties via written request to the authors.

## Code Availability

Software used in this study is commercially available from MathWorks, Natick, Massachusetts, United States
